# FURIN and placental syncytialisation: a cautionary tale

**DOI:** 10.1038/s41419-021-03898-z

**Published:** 2021-06-21

**Authors:** Saije K. Morosin, Sarah J. Delforce, Celine Corbisier de Meaultsart, Eugenie R. Lumbers, Kirsty G. Pringle

**Affiliations:** School of Biomedical Sciences and Pharmacy, Priority Research Centre for Reproductive Science, Pregnancy and Reproduction Program, Hunter Medical Research Institute, University of Newcastle, Newcastle, NSW Australia

**Keywords:** Proteases, Differentiation

## Abstract

FURIN is a pro-protein convertase previously shown to be important for placental syncytialisation (Zhou et al. [1]), a process of cell fusion whereby placental cytotrophoblast cells fuse to form a multinucleated syncytium. This finding has been broadly accepted however, we have evidence suggesting the contrary. Spontaneously syncytialising term primary human trophoblast cells and BeWo choriocarcinoma cells were treated with either *FURIN* siRNA or negative control siRNA or the protease inhibitor, DEC-RVKR-CMK, or vehicle. Cells were then left to either spontaneously syncytialise (primary trophoblasts) or were induced to syncytialise with forskolin (BeWo). Effects on syncytialisation were measured by determining human chorionic gonadotrophin secretion and E-cadherin protein levels. We showed that FURIN is not important for syncytialisation in either cell type. However, in primary trophoblasts another protease also inhibited by DEC-RVKR-CMK, may be involved. Our results directly contrast with those published by Zhou et al. Zhou et al. however, used first trimester villous explants to study syncytialisation, and we used term primary trophoblasts. Therefore, we suggest that FURIN may be involved in syncytialisation of first trimester trophoblasts, but not term trophoblasts. What is more concerning is that our results using BeWo cells do not agree with their results, even though for the most part, we used the same experimental design. It is unclear why these experiments yielded different results, however we wanted to draw attention to simple differences in measuring syncytialisation or flaws in method reporting (including omission of cell line source and passage numbers, siRNA concentration and protein molecular weights) and choice of immunoblot loading controls, that could impact on experimental outcomes. Our study shows that careful reporting of methods by authors and thorough scrutiny by referees are vital. Furthermore, a universal benchmark for measuring syncytialisation is required so that various studies of syncytialisation can be validated.

## Introduction

Placental trophoblast syncytialisation is the process of cell fusion whereby the cytotrophoblast cells fuse to form a multinucleated syncytiotrophoblast. The syncytiotrophoblast is vital for regulating transport of nutrients and wastes between mother and baby, whilst also secreting essential pregnancy hormones. Hence, understanding how this occurs is pivotal to understanding placentation.

There are two major cell models used when exploring syncytialisation in vitro. These include spontaneously syncytialising primary human trophoblast cells, isolated directly from human placentae, and a forskolin-induced model of syncytialisation using BeWo choriocarcinoma cells. Syncytialisation can be measured in both cell models via secretion of human chorionic gonadotropin (hCG) and assessment of E-cadherin levels [2, 3].

FURIN is a pro-protein convertase subtilisin/kexin (PCSK; aka PCSK3) responsible for cleavage and activation of a number of pro-proteins [4]. FURIN cleaves important fusogenic genes Syncytin 1 and 2 [5] and it is currently understood that FURIN is important for placental trophoblast syncytialisation [1]. Zhou et al. showed that FURIN is increased as primary trophoblasts and BeWo choriocarcinoma cells syncytialise and that knockdown of FURIN using an siRNA or inhibition using the broad protease inhibitor DEC-RVKR-CMK (which inhibits the activity of all PCSKs 1-7 [6, 7]), significantly inhibited syncytialisation in first trimester placental explants and in forskolin-treated BeWo choriocarcinoma cells [1, 8].

We have previously reported that term primary trophoblasts show decreased FURIN mRNA and protein levels with syncytialisation [9]; results that are opposite to those of Zhou et al. Therefore, further investigation into the role of FURIN in syncytialisation of primary trophoblast cells is required.

We suggest that while FURIN may be important for syncytialisation in the first trimester, it is not involved in syncytialisation of term primary trophoblast cells nor BeWo choriocarcinoma cells. Our data show that care needs to be taken in interpreting experimental findings relating to the role of FURIN in human placental syncytialisation.

## Methods

### Ethical approval

Ethics approval was obtained from the University of Newcastle Human Research Ethics Committee (H-382-0602 and H-2020-0398) and the Hunter New England Human Ethics Committee (02/06/12/3.13) to carry out this work. All placental tissues were obtained with written and informed consent.

### Primary trophoblast cell culture

Non-labouring term human placentae were donated with informed consent by women delivering uncomplicated singleton pregnancies via elective caesarean section at the John Hunter Hospital, Newcastle, Australia. Exclusion criteria are as previously described [10].

Primary trophoblast isolations were performed as previously described by Kaitu’u-lino et al. within 30 min of delivery and included performing negative selection using a CD9 antibody (MAB1880, R&D Systems, MN, USA), ensuring a pure population of trophoblast cells [11]. Cells were plated as previously described in Morosin et al. [10]. Briefly, 12 or 24-well plates with coverslips inserted were coated with fibronectin (10 μg/mL), prior to trophoblast cell seeding at 800,000 cells/well. Trophoblasts were maintained in 1× high-glucose Dulbecco’s Modified Eagles Medium (DMEM-HG; Hyclone, UT, USA) supplemented with 10% heat inactivated fetal bovine serum (FBS; Bovogen Biologicals, Vic, Australia), 2 mM L-glutamine (Gibco, CA, USA) and 1% antibiotic-antimycotic (Gibco) in 5% CO_2_ in room air at 37 °C. Primary trophoblast cell cultures were probed with Cytokeratin 7 (CK7) using immunocytochemistry (ICC) to assess the purity of the cell population. Assessment of CK7 levels indicated that 93% of the cells were trophoblasts (*N* = 5 placentae; data not shown).

### BeWo Choriocarcinoma cell culture

BeWo choriocarcinoma cells (American Type Culture Collection, CCL-98, USA; passages 7–18) were plated at 200,000 cells/well in six-well plates. Cells were cultured as above, however 10% non-heat inactivated FBS was used. For ICC experiments coverslips were inserted and coated with 0.1% gelatin prior to cell seeding.

### siRNA transfection and DEC-RVKR-CMK treatment

Twenty-four hours after plating cells underwent either:

- siRNA transfection. In primary trophoblasts this was performed as previously described in Morosin et al. [9, 10] using 10 nM *FURIN* siRNA (HSS107545, Life Technologies, CA, USA) or negative control siRNAs (medium GC; Invitrogen). In BeWo cells 125 nM *FURIN* siRNA was used. siRNA was combined with lipofectamine (Invitrogen), Opti-MEM (Gibco) and the appropriate complete culture medium (without antibiotics). Cells treated with lipofectamine and opti-MEM alone were also included as non-transfected controls.
  - Primary trophoblast cells were cultured for 72 h, collected and snap frozen in TRIzol reagent (Invitrogen, USA).
  - 24 h after transfection BeWo cells were treated with 100 μM forskolin (Sigma-Aldrich, USA) or vehicle (dimethyl sulphoxide (DMSO); UNIVAR, USA) to induce syncytialisation; 48 h after forskolin treatment BeWo cells and supernatant were collected and snap frozen.
  OR
- DEC-RVKR-CMK treatment. Primary trophoblast and BeWo cells were treated with 50 μM FURIN/broad protease inhibitor (DEC-RVKR-CMK; Merck, USA) or vehicle (DMSO). BeWo cells were simultaneously treated with 100 μM forskolin or vehicle (DMSO). Cells were incubated for 48 h for syncytialisation to occur and collected in TRIzol reagent.

### Protein extraction

Protein from all primary trophoblast experiments and BeWo DEC-RVKR-CMK experiments were isolated using TRIzol reagent according to the manufacturer’s instructions. However, protein pellets were dissolved in a solubilisation buffer which contained 20 nM EDTA (Invitrogen), 140 mM sodium chloride (NaCl; VWR international), 5% sodium dodecyl sulfate (SDS; Sigma-Aldrich), 100 mM tris-HCL (Invitrogen), 1 mM sodium orthovanadate (Sigma-Aldrich) and a complete mini protease inhibitor cocktail tablet (Sigma-Aldrich), as described by Morosin et al. [10].

Protein from BeWo siRNA experiments was isolated using a radio immuno-precipitation assay (RIPA) method, as described previously [12]. Briefly, 100 μl RIPA buffer (containing 50 mM tris-HCL, 150 mM NaCl, 1 mM EDTA, 1% NP-40 (Sigma-Aldrich), 0.5% Sodium Deoxycholate (Sigma-Aldrich), 1 mM sodium orthovanadate, a complete mini protease inhibitor cocktail tablet and 1 μl of 100 nM phenylmethylsulfonyl fluoride (PMSF; Sigma-Aldrich)) was added to each sample and vortexed. Samples were then vortexed every 10 min for 30 min before being centrifuged at 13,000 RPM at 4 °C for 10 min. Supernatants were then collected and stored at −80 °C.

Protein concentrations were measured using a bicinchoninic acid (BCA) assay (ThermoFisher Scientific) according to the manufacturer’s instructions.

### Measurement of syncytialisation markers CDH1 (E-cadherin) and hCG

Protein lysates underwent immunoblotting and were probed for CDH1 (E-cadherin) and ACTB (β-actin) as previously described [10]. Full length immunoblot images are included in Supplementary Fig. 1. Briefly, 5 μg of total protein was separated on 4–12% Bis-Tris, precast NuPAGE Novex gels (Life Technologies) using electrophoresis and, using the Novex^®^ NuPAGE^®^ SDS-PAGE Gel System (Thermofisher), were then transferred onto polyvinylidene fluoride (PVDF) membrane. Membranes were first blocked in 5% skim milk and 5% bovine serum albumin (BSA) in tris buffered saline containing 20% Tween 20 (TBST) overnight at 4 °C. Subsequently, membranes were probed for CDH1 at 0.1 μg/mL in 5% skim milk in TBST (ab1416; abcam, UK) for 2 h at room temperature. Membranes then underwent a series of washes in TBST and TBS before incubation with an anti-mouse HRP secondary antibody at 0.05 μg/mL in 1% skim milk in TBST (7076; Cell Signalling; USA) for 1 h at room temperature. Detection was performed using Amersham ECL and an Amersham Imager 600 (GE Healthcare Life Sciences; USA). All membranes were then stripped using 0.2 M sodium hydroxide and re-probed for ACTB (0.04 μg/mL; ab8227; abcam) using an anti-rabbit HRP secondary antibody (0.2 μg/mL;12-348; Millipore; USA).

Densitometry was performed using Amersham Imager 600 software. Data are presented as a ratio of CDH1/ACTB. Samples were run in duplicate and results were averaged and corrected to an internal control included on every blot.

Fixed cells were probed for CDH1 (E-cadherin) using ICC as previously described [10]. Cells were permeabilised with 0.1% Triton X-100 in phosphate buffered saline (PBS; Bio-Rad Australia). Cells were then washed with PBS and blocked using 1% BSA/PBS for 1 h at room temperature. Probing for CDH1 then took place using ab1416 at 0.4 μg/mL diluted in 0.1% BSA/PBS, for 2 h at room temperature. Cells were then washed with PBS and incubated with a secondary antibody at 1.3 μg/mL diluted in 0.1% BSA/PBS (Alexa Flour 488 goat anti-mouse IgG; A-11029; Thermofisher; USA) for 1 h at room temperature away from light. Note that negative controls were included, which involved incubating cells in BSA/PBS in the absence of either the primary antibody alone or both the primary and secondary antibody. Coverslips were then mounted onto microscope slides using prolong diamond anti-fade mountant with DAPI (P36962; Thermofisher Scientific) and stored at 4 °C. Slides were imaged using a Nikon C2 confocal microscope at ×40 magnification.

Analysis was performed using Image J cell counting software. Syncytialisation was determined by dividing the total number of nuclei within a syncytium by the total number of nuclei present, and converted to a percentage by multiplying by 100, as previously described in Morosin et al. [10].

Secreted hCG was measured via Enzyme linked immunosorbent assay (Thermofisher Scientific) according to the manufacturer’s instructions and previously described [10]. A SPECTROstar^nano^ microplate reader was used to measure optical densities and samples were normalised to their media blank to remove background interference. For primary trophoblasts experiments the intra-assay coefficient of variance (CV) was 4.2% and the inter-assay CV was 12.9%. For BeWo intra-assay CV was 2.4% and inter-assay CV was 12%.

### Statistics

All experiments were plated in triplicate wells except for ICC experiments, which were plated in single wells but imaged in three different areas per well. All experiments were repeated on at least three separate occasions (BeWo: *N* = 3; trophoblast: *N* = 4–5 placentae). GraphPad prism version 8.0 was used for all statistical analyses and significance set at *P* < 0.05. Nonparametric tests were used as the data was not normally distributed. Mann–Whitney statistical tests were used for primary trophoblast statistical analyses and two-way analysis of variance tests with Sidak’s multiple comparisons test were used for BeWo statistical analysis.

## Results

### The effect of *FURIN* siRNA knockdown and DEC-RVKR-CMK treatment on spontaneous syncytialisation of term primary human trophoblasts and forskolin-induced syncytialisation of BeWo choriocarcinoma cells

**Fig. 1:**
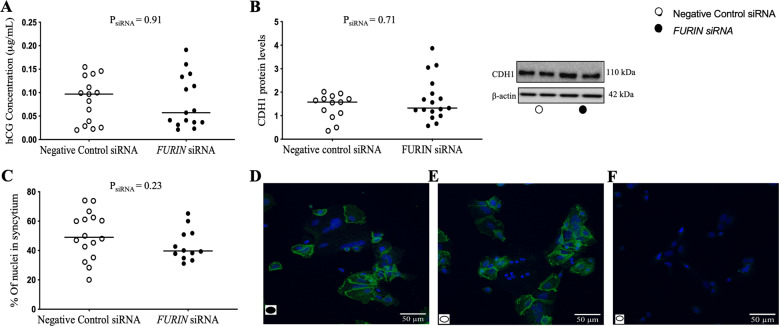
*FURIN* siRNA did not affect syncytialisation of term human primary trophoblast cells. Trophoblasts were treated with *FURIN* siRNA and left to syncytialise for 72 h. *FURIN* siRNA had no effect on (**A**) hCG secretion, (**B**) E-cadherin (CDH1) protein levels or (**C**) the percent of nuclei in syncytia, compared with negative control siRNA. Representative images in (**D**, **E**) depict cells stained for CDH1 (green) and counterstained with DAPI (blue). **F** is a negative control image omitting the primary antibody, controlling for non-specific secondary antibody binding. β-actin was used as a loading control in immunoblots. Data are presented as the median with effects of *FURIN* siRNA (P_siRNA_) noted. *N* = 5 placentae in triplicate. For immunocytochemistry only: *N* = 5 in singlicate with three images/well.

**Fig. 2:**
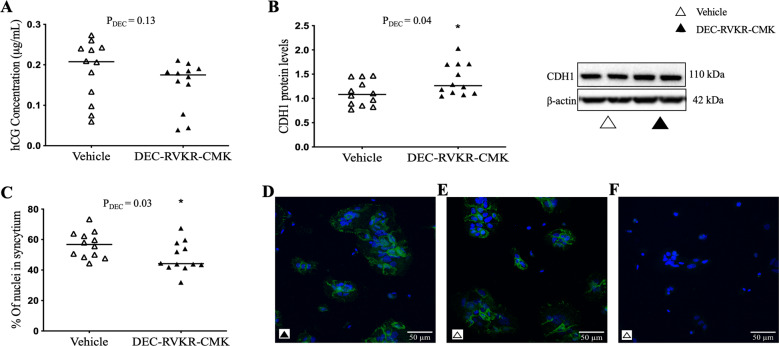
DEC-RVKR-CMK did not affect syncytialisation of term human primary trophoblast cells. Trophoblast cells were treated with 50 μM of the protease inhibitor DEC-RVKR-CMK and left to syncytialise for 48 h. DEC-RVKR-CMK had no effect on (**A**) hCG secretion, significantly increased (**B**) CDH1 protein levels, but did not affect (**C**) the percent of nuclei in syncytia. Representative images in (**D**, **E**) depict cells stained for CDH1 (green) and counterstained with DAPI (blue). **F** Is a negative control image omitting the primary antibody, controlling for non-specific secondary antibody binding. β-actin was used as a loading control in immunoblots. Asterisk (*) denotes significant difference to vehicle control. Data are presented as the median with effects of DEC-RVKR-CMK (P_DEC_) noted. *N* = 4 placentae in triplicate. For immunocytochemistry only: *N* = 4 placentae in singlicate with three images/well.

Primary trophoblasts spontaneously syncytialised in culture [10] and *FURIN* siRNA transfection successfully decreased FURIN mRNA expression by 4.2-fold and protein levels by 3.9-fold at 72 h (Supplementary Fig. 2). *FURIN* siRNA also successfully decreased FURIN enzyme activity [9]. It should be noted that these samples are a subset of those used in Morosin et al. [9, 10]. *FURIN* siRNA had no effect on hCG secretion (Fig. 1A), E-cadherin (CDH1) protein levels (Fig. 1B) or the percent of nuclei within syncytia (Fig. 1C–F). Furthermore, while treatment with the broad protease inhibitor, DEC-RVKR-CMK, decreased FURIN enzyme activity [9], significantly increased CDH1 protein levels (Fig. 2B; *P* = 0.04), and reduced the percent of nuclei in syncytia (Fig. 2C–F; *P* = 0.03), it had no effect on hCG secretion (Fig. 2A).

**Fig. 3:**
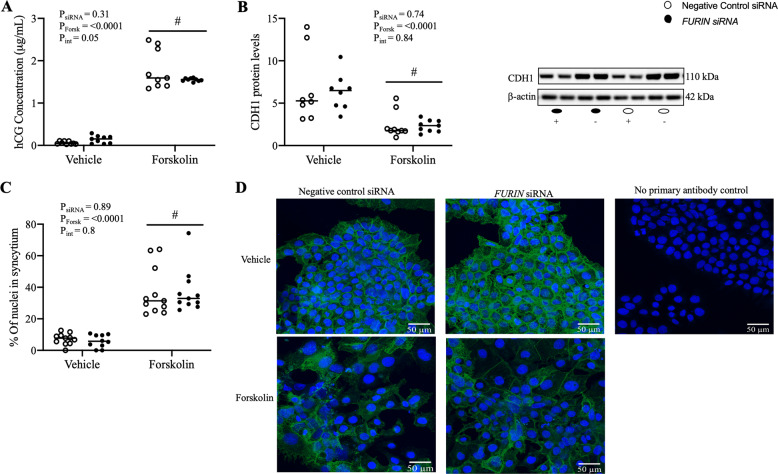
FURIN knockdown had no effect on forskolin-induced syncytialisation of BeWo choriocarcinoma cells. BeWo cells were treated with *FURIN* siRNA prior to being induced to syncytialise with forskolin. Forskolin treatment successfully (**A**) increased hCG secretion, (**B**) decreased E-cadherin (CDH1) protein levels and (**C**) increased the percent of nuclei in syncytia (as determined by CDH1 immunostaining). *FURIN* siRNA knockdown had no effect on (**A**) hCG secretion, (**B**) E-cadherin (CDH1) protein levels or (**C**) the percent of nuclei in syncytia, respectively. Representative images in (**D**) depict cells stained for CDH1 (green) and counterstained with DAPI (blue). No primary antibody control images omitting the primary antibody are included, controlling for non-specific secondary antibody binding. Representative blots are shown where β-actin was used as a loading control. # Denotes significant difference to the vehicle control. Data are presented as the median with effects of Forskolin (P_Forsk_), *FURIN* siRNA (P_siRNA_), or the interaction between these parameters (P_Int_) noted. *N* = 3 experiments in triplicate. For immunocytochemistry only: *N* = 3 in singlicate (three images/well).

**Fig. 4:**
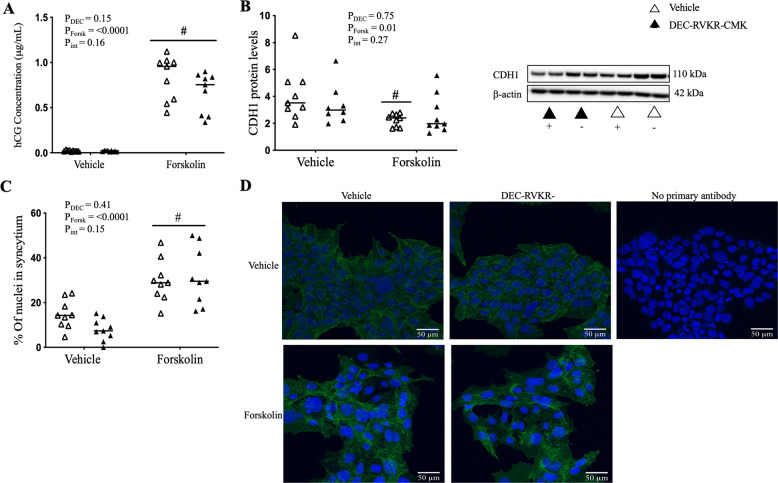
DEC-RVKR-CMK had no effect on forskolin-induced syncytialisation of BeWo choriocarcinoma cells. BeWo cells were simultaneously treated with DEC-RVKR-CMK and forskolin and left to syncytialise. Forskolin treatment successfully induced syncytialisation, this is depicted by increased (**A**) hCG secretion, decreased (**B**) E-cadherin (CDH1) protein levels and increased (**C**) percent of nuclei in syncytia (as determined by CDH1 immunostaining). DEC-RVKR-CMK treatment had no effect on (**A**) hCG secretion, (**B**) E-cadherin (CDH1) protein levels or (**C**) the percent of nuclei in syncytia (as determined by CDH1 immunostaining), respectively. Representative images in (**D**) depict cells stained for CDH1 (green) and counterstained with DAPI (blue). No primary antibody control images omitting the primary antibody are included, controlling for non-specific secondary antibody binding. Representative blots are shown where β-actin was used as a loading control. # Denotes significant difference to the forskolin vehicle control. Data are presented as the median with effects of Forskolin (P_Forsk_), DEC-RVKR-CMK (P_DEC_) or the interaction between these parameters (P_Int_) noted. *N* = 3 experiments in triplicate. For immunocytochemistry only: *N* = 3 in singlicate (three images/well).

In BeWo cells forskolin successfully induced syncytialisation, as determined by increased hCG secretion (Figs. 3 and 4A), decreased E-cadherin protein levels (Figs. 3 and 4B) and increased percent of nuclei in syncytia (Figs. 3 and 4C) in the forskolin-treated group compared to vehicle. *FURIN* siRNA successfully decreased FURIN mRNA expression by 2.5 and 2.2-fold and protein by 2.4 and 2.1-fold, in vehicle and forskolin groups, respectively (Supplementary Fig. 2). Additionally, both *FURIN* siRNA and DEC-RVKR-CMK treatment inhibited FURIN enzyme activity (Supplementary Fig. 3). Neither *FURIN* siRNA nor treatment with DEC-RVKR-CMK affected hCG secretion, CDH1 protein levels or the percent of nuclei in syncytia, in either forskolin or vehicle treated groups (Figs. 3 and 4*,* respectively).

## Discussion

We have shown that FURIN does not play a role in spontaneous syncytialisation of term human primary trophoblast cells nor in forskolin-induced syncytialisation of BeWo choriocarcinoma cells. Our data challenge the current dogma surrounding the role of FURIN in the placenta.

We have shown that *FURIN* siRNA knockdown did not effect spontaneous syncytialisation of term primary trophoblast cells (Fig. 1). However, DEC-RVKR-CMK significantly increased CDH1 (E-cadherin) protein levels and reduced the number of nuclei within a CDH1 boundary (Fig. 2). This indicates that FURIN is not involved in term trophoblast syncytialisation, but that a protease also inhibited by the broad protease inhibitor, DEC-RVKR-CMK, is involved. This finding is in contrast to findings by Zhou et al. who, while also showing that DEC-RVKR-CMK reduced syncytiotrophoblast formation, also showed that *FURIN* siRNA reduced syncytiotrophoblast formation in first trimester placental explants [1].

There are, however, clear differences in the syncytialisation models and methods used in the two studies. First, Zhou et al. used placental explants from first trimester placentae, whereas we used term primary trophoblast cells isolated from term placentae. This could explain our different results. Not only do villous explants contain a number of different placental cell types that could affect FURIN activity, but an earlier study by Zhou et al. has shown that FURIN expression is higher in first trimester placenta [13]. Therefore, first trimester placental explants are also likely to have higher FURIN expression than term primary trophoblast cells. Together, this suggests that while FURIN is involved in first trimester trophoblast syncytialisation; it is not involved in term trophoblast syncytialisation.

It is also possible that differences in experimental oxygen tensions could have affected the outcomes of the two studies. Zhou et al. cultured first trimester explants at the physiological placental oxygen tension of 8%, whereas we cultured term primary trophoblast cells at standard culture conditions (20% O_2_). Interestingly, hypoxia can increase FURIN levels in HepG2 human hepatoma cells through hypoxia-inducible factor 1 (HIF1) [14]. Hence, culture in 8% O_2_ may have increased FURIN levels in Zhou et al.’s experiments, suggesting that there is involvement of FURIN in syncytialisation. In a similar manner, since HIF1 is stabilised at low oxygen tensions and has been shown to subsequently regulate FURIN [14], this may indicate why the same increase in FURIN expression with syncytialisation, was not seen in cultures incubated in 20% O_2_. It may also be the reason why FURIN levels are increased in first trimester placentae compared to term. However, previous studies in 9% oxygen have reported that hypoxia impairs term trophoblast syncytialisation [15]. Therefore further investigation into the effects of oxygen tension on FURIN expression and syncytialisation in the placenta is required.

Finally, we have estimated syncytialisation differently to Zhou et al. Zhou et al. used the syncytiotrophoblast regeneration rate as a sole indicator of syncytialisation in first trimester explants. This is calculated by immunostaining placental villous explants for hCG and cytokeratin 7 (CK7) and counting the number of nuclei in the hCG positive syncytiotrophoblast layer and dividing it by the total number of CK7 positive cytotrophoblast cells present. This measure is not widely used or validated in the literature. We have, on the other hand, used three different markers to measure syncytialisation including hCG secretion, e-cadherin protein levels, and calculation of the percent of nuclei within syncytia (via e-cadherin immunostaining). These methods are all validated in the literature [2, 3] and widely used to assess syncytialisation [15–17]. Additionally, first trimester placental explants contain a number of different cell types that could impact on trophoblast syncytialisation. These include placental mesenchymal stem/stromal cells and Hofbauer cells, which can secrete cytokines and growth factors that are able to affect trophoblast differentiation [18, 19]. Of significance, Hofbauer cells (of which there are more in the first trimester compared with the third trimester placenta [20]) can stimulate hCG production suggesting that they can stimulate trophoblast differentiation [19].

We were also unable to reproduce results by Zhou et al. using BeWo choriocarcinoma cells. First, there was no change in FURIN mRNA expression or protein levels with forskolin-induced syncytialisation (see Supplementary Fig. 2), whereas Zhou et al. showed an increase [1, 8]. The normalisation of protein data to GAPDH may account for this difference as a clear decrease in GAPDH protein levels is seen in BeWo cells treated with forskolin (Zhou et al. Fig. 1 [1]). This could have impacted the quantification of furin protein levels causing false positive results. They also used *GAPDH* mRNA to normalise *FURIN* mRNA expression [8], and this may also have impacted on the quantification of *FURIN* mRNA levels. This highlights the importance of selecting appropriate housekeeping genes and proteins.

Second, although Zhou et al. showed that *FURIN* siRNA and DEC-RVKR-CMK inhibited forskolin-induced syncytialisation of BeWo cells [1], we found that they had no effect (Figs. 3, 4). The two studies are virtually identical in terms of study design; both used BeWo cells and induced syncytialisation with 100 μM forskolin over 48 h, and both used the same oxygen tension, eliminating any potential effects of oxygen on FURIN expression (as described for primary cells above). As well, we both used a *FURIN* siRNA or the protease inhibitor DEC-RVKR-CMK. We did however use 50 μM of DEC-RVKR-CMK and Zhou et al. used 25 μM. We would have expected our higher dose would, if anything, have accentuated the effects seen by Zhou et al. instead it had no effect. It is difficult to say why such opposite effects were obtained.

One possibility is that syncytialisation was measured slightly differently between the two studies. Zhou et al. measured hCG in BeWo cell lysates; we measured secreted hCG. There is no evidence to show that intracellular hCG levels parallel that of secreted hCG, but it is the secreted form of hCG that acts on the luteinising hormone/chorionic gonadotrophin receptor (LH/CGR) located on the cell plasma membrane, regulating the rate of syncytialisation and its own secretion [21, 22]. For these reasons we chose to measure secreted hCG; we believe it is a more accurate measure of syncytialisation.

More information could have been provided by Zhou et al. that could help explain our different results. Experiments should be sufficiently robust so that findings are easily reproduced by other laboratories, but lack of information about the experimental methods makes reproduction of data difficult. For example, it is not clear as to the source or passage number of the BeWo cells used by Zhou et al. [1]. High passage numbers can change cell characteristics including; morphology, protein expression and transfection efficiency [23–25]. Furthermore, the concentration of *FURIN* siRNA used in BeWo experiments was not listed, nor were the molecular weights of FURIN, hCG and GAPDH proteins. These factors could account for the differences between studies.

The overall common link that does potentially explain our different findings is that the methods for quantitating syncytialisation are different in studies involving both primary tissue/cells and BeWo cells. This highlights the need for universal guidelines or benchmarks for measuring syncytialisation, which could avoid some of the issues raised in this manuscript and ensure that consistent results are obtained by different laboratories.

To conclude, our results challenge the current research surrounding FURIN in placental trophoblast syncytialisation. We have shown that FURIN is not involved in spontaneous syncytialisation of term primary trophoblasts or forskolin-induced syncytialisation of BeWo choriocarcinoma cells. Additionally, we have shown that a protease, other than FURIN, that is inhibited by the broad protease inhibitor, DEC-RVKR-CMK, is involved. However, the clear difference in experimental models using primary tissue/cells means that further experimentation is required in order to definitively determine if FURIN plays a role in syncytialisation. These may include repetition of this study using our technology in first trimester primary trophoblast cells and/or performing experiments using FURIN overexpression in both primary trophoblast and BeWo cells. Our study highlights the need for careful reporting of methods by authors and careful assessment of manuscripts by the research community, before accepting findings. It also reveals the need for a universal benchmark for measuring syncytialisation.

## Supplementary information

Supplementary Figure 1

Supplementary Figure 2

Supplementary Figure 3
