# Supplementary material for: FURIN and placental syncytialisation: a cautionary tale
Source: Cell Death Dis. 2021 Jun 21;12(7):635. doi: 10.1038/s41419-021-03898-z (PMC8217546; doi:10.1038/s41419-021-03898-z)
Supplement: Supplementary file 1 — Supplementary Figure 1 [file 41419_2021_3898_MOESM1_ESM.docx]

*Supplementary Figure 1: Representative full-length immunoblot images of E-cadherin (CDH1) and β-actin densitometry for primary trophoblast and BeWo cell lysates.* In primary trophoblasts (**A**) CDH1 was detected as a clear singular band at 110 kDa and (**B**) β-actin was also detected as a clear singular band at 42 kDa. The same was true for BeWo choriocarcinoma cell samples, with (**C**) CDH1 protein depicted at 110 kDa and (**D**) β-actin protein depicted at 42 kDa. The results of samples treated with an alternate siRNA were not included within this paper. IC is the internal control that was loaded on every gel. **A/B** are full length blots of the representative blot in Figure 1B and **C/D** are full length blots of the representative blot in Figure 3B. The red boxes depict bands shown in the representative blot. + represents samples treated with forskolin. – represents vehicle treated samples.
