# Supplementary material for: FURIN and placental syncytialisation: a cautionary tale
Source: Cell Death Dis. 2021 Jun 21;12(7):635. doi: 10.1038/s41419-021-03898-z (PMC8217546; doi:10.1038/s41419-021-03898-z)
Supplement: Supplementary file 2 — Supplementary Figure 2 [file 41419_2021_3898_MOESM2_ESM.docx]

*Supplementary Figure 2: FURIN siRNA knockdown was successful in both primary trophoblast and BeWo choriocarcinoma cells.* Primary trophoblast and BeWo choriocarcinoma cells were treated with *FURIN* siRNA to knockdown *FURIN* mRNA expression and protein levels. Primary trophoblasts were then left to spontaneously syncytialise for 72 h, while BeWo cells were induced to syncytialise with Forskolin. In primary trophoblast cells *FURIN* siRNA treatment significantly decreased both (**A**) *FURIN* mRNA expression and (**B**) FURIN protein levels at 72 h. FURIN mRNA and protein detection were performed as described previously^1^. In BeWo cells, forskolin treatment did not affect either (**C**) *FURIN* mRNA expression or (**D**) FURIN protein levels (multiple comparisons analysis shows P=0.06 and P=0.71 for negative control and *FURIN* siRNA treatment groups respectively). However, *FURIN* siRNA significantly decreased (**C**) *FURIN* mRNA expression (both P=0.004) and (**D**) FURIN protein levels (P<0.0001 and P=0.002), in both vehicle and forskolin treated groups respectively. FURIN mRNA and protein detection were performed as described previously^1^, except RNA was extracted using an RNeasy-mini Kit (Qiagen, Venlo, Netherlands) according to the manufacturers instructions and protein extracted using a radio immuno-precipitation assay (RIPA) method, as described previously^2^. β-actin was used as a loading control in the representative immunoblots. Primary trophoblast experiments N=5 placentae in triplicate. BeWo experiments N=3 experiments in triplicate.

*References:*

1 Morosin, S. K., Delforce, S. J., Lumbers, E. R. & Pringle, K. G. Cleavage of the soluble (pro)renin receptor (sATP6AP2) in the placenta. *Placenta* **101**, 49-56, doi:<https://doi.org/10.1016/j.placenta.2020.08.019> (2020).

2 Delforce, S. J., Lumbers, E. R., Morosin, S. K., Wang, Y. & Pringle, K. G. The Angiotensin II type 1 receptor mediates the effects of low oxygen on early placental angiogenesis. *Placenta* **75**, 54-61, doi:<https://doi.org/10.1016/j.placenta.2018.12.001> (2019).
