# Supplementary material for: FURIN and placental syncytialisation: a cautionary tale
Source: Cell Death Dis. 2021 Jun 21;12(7):635. doi: 10.1038/s41419-021-03898-z (PMC8217546; doi:10.1038/s41419-021-03898-z)
Supplement: Supplementary file 3 — Supplementary Figure 3 [file 41419_2021_3898_MOESM3_ESM.docx]

*Supplementary Figure 3: Treatment of BeWo choriocarcinoma cells with either FURIN siRNA or the proprotein convertase inhibitor DEC-RVKR-CMK inhibited FURIN enzyme activity*. FURIN is known to cleave the insulin-like growth factor 1 receptor-β (IGF1R-β) into its active form (Khatib et.al., J Biol Chem. 2001). Hence to determine if *FURIN* siRNA and DEC-RVKR-CMK effected FURIN enzyme activity, levels of pro and active IGF1R-β were assessed. Treatment with both *FURIN* siRNA and DEC-RVKR-CMK were associated with increased pro-IGF1R-β (**A** and **C**) and decreased active IGF1R-β (**B** and **D**), regardless of forskolin treatment. Representative densitometry shows a ponceau stain, which was used as a loading control. Data are presented as mean ± SEM. N=1 experiment in triplicate. Immunoblots were performed using an IGF1R-β primary antibody (Cell signaling; 9750S) and an anti-rabbit HRP secondary antibody (Merk; 12-348) using methods previously described in Morosin et.al., 2020^1,2^.

*References:*

1 Morosin, S. K., Delforce, S. J., Lumbers, E. R. & Pringle, K. P. The (pro)renin receptor (ATP6AP2) does not play a role in syncytialisation of term human primary trophoblast cells. *Placenta* **97**, 89-94, doi:<https://doi.org/10.1016/j.placenta.2020.05.009> (2020).

2 Morosin, S. K., Delforce, S. J., Lumbers, E. R. & Pringle, K. G. Cleavage of the soluble (pro)renin receptor (sATP6AP2) in the placenta. *Placenta* **101**, 49-56, doi:<https://doi.org/10.1016/j.placenta.2020.08.019> (2020).
